# Structural and functional characterisation of the Crimean-Congo haemorrhagic fever virus RNA dependent RNA polymerase

**DOI:** 10.1038/s41467-026-74113-w

**Published:** 2026-06-09

**Authors:** Adrian Deng, Rory Cunnison, Loïc Carrique, Franziska Günl, Els Pardon, Jan Steyaert, David Nguyen Duc, Jonathan M. Grimes, Nicole Robb, Jeremy R. Keown

**Affiliations:** 1 School of Life Sciences, University of Warwick, Coventry, UK; 2 Warwick Medical School, University of Warwick, Coventry, UK; 3 Division of Structural Biology, Centre for Human Genetics, University of Oxford, Oxford, UK; 4 Sir William Dunn School of Pathology, University of Oxford, Oxford, UK; 5 Structural Biology Brussels, Vrije Universiteit Brussel, VUB, Brussels, Belgium; 6 VIB-VUB Center for Structural Biology, VIB, Brussels, Belgium

**Keywords:** Virology, Cryoelectron microscopy

## Abstract

Crimean-Congo Haemorrhagic Fever Virus (CCHFV) is found across Africa, Asia, and the Middle East where it can cause Haemorrhagic outbreaks with high case fatality rates. Central to the viral life cycle is the viral L-protein, a crucial and multifunctional protein which both transcribes and replicates the viral genome. Here, we present the cryoEM structures of an RNA free and a 5′ promoter bound complex, describing the core catalytic RNA-dependent RNA polymerase (RdRp). We observe an RdRp that is substantially larger than related L-proteins and contains domain insertions unique to the nairovirus family. The 5′ RNA promoter is found in a tight RNA hairpin stabilised by a single base pair, with 5′ binding triggering the closure of protein over the RNA. Functional analysis of the endonuclease and RdRp activities reveals an enzyme which is capable of both activities and demonstrate RdRp inhibition by known antiviral nucleosides. These data advance our understanding of the molecular mechanisms behind genome replication and transcription, that will help inform future antiviral development.

## Abstract (web summary)

Authors present CryoEM structures of the Crimean-Congo haemorrhagic fever virus L-protein RdRp core bound to the 5′ genome end, develop in vitro functional assays, and demonstrate their utility for antiviral screening.

## Introduction

Crimean-Congo haemorrhagic fever virus (CCHFV, *Orthonairovirus haemorrhagiae*) belongs to the family *Nairoviridae* within the *Bunyaviricetes* class which contains several emerging human and animal pathogens^1^. The Nairovirus family contains over sixty species, with CCHFV causing the majority of human infections and a smaller number of infections being caused by Nairobi sheep disease virus (NSDV)^2^. CCHFV is a tick-borne arbovirus with a broad geographic distribution that includes Africa, the Middle East, Asia and, more recently, southern and eastern Europe^3^. Human infection can lead to haemorrhagic disease with case fatality rates exceeding 30%^4^, although the true incidence is likely underestimated due to a high proportion of subclinical infections. The virus is primarily transmitted by ticks from the *Hyalomma* genus^5^, but human infections can occur through contact with infected animal material.

CCHFV genome organisation is similar to other *Bunyaviricetes*, comprising a negative sense segmented RNA genome^1^. The genome segments are called small (S), medium (M), and large (L). The S segment encodes the viral nucleoprotein (NP) and in an opposite sense open reading frame the small nonstructural (NSs) protein^6^. The M segment is more complex than typical *Bunyaviricetes*, encoding not only the viral glycoproteins (Gn and Gc) which perform receptor entry and binding, but also the GP160/85 precursor protein, and a medium nonstructural protein (NSm). The L segment encodes the CCHFV L-protein (CCHFV-L). In addition to the coding region, each segment, possesses short untranslated regions at the 5′ and 3′ termini^7^.

Each negative sense RNA segment is packaged into viral ribonucleoprotein (vRNP) complexes that contain a single RNA segment, many copies of NP which bind and protect the RNA, and a single copy of CCHFV-L. The vRNP are the structural and functional units of genome transcription and replication with CCHFV-L providing the enzymatic activities to perform these processes. Transcription initiates with ‘cap-snatching’, a process where CCHFV-L binds host mRNA caps, cleaves a short capped RNA primer, which is then annealed to the 3′ termini of the genome and extended. Genome replication proceeds in a two-step process. First a positive sense complimentary RNA (cRNA) intermediate is synthesised, which is used as a template to produce new vRNA copies in the second step^8^.

Recently L-protein structures have been elucidated for several members of the *Bunyaviricetes* class. These include members of the *Phenuiviridae*^9–12^, *Arenaviridae*^13,14^, *Peribunyaviridae*^15–17^, *Tospoviridae*^18^, and *Hantaviridae*^19–22^. The L-proteins from these viral families are single polypeptides of approximately 250 kDa that contain endonuclease, RNA-dependent RNA polymerase (RdRp), and cap-binding domains^8^. These structures present snapshots of L-proteins without RNA and in conformations related to discrete stages of replication and transcription, mapping the location of the 5′ promoter binding site and the 3′ RNA in several positions.

In contrast to the properties of the viral L-proteins described above, CCHFV-L is almost twice the size at approximately 450 kDa. The N-terminal 169 amino acids of the polypeptide encode an Ovarian tumour like (OTU) protease domain for which several structures have been determined alone and in complex with the interferon-stimulated gene 15 (ISG15) or ubiquitin^23–25^. Residues 587-895 in CCHFV-L encode a putative endonuclease domain with D693 as a key active site residue as identified by virus like particle assays^26^. In vitro analyses of the purified endonuclease domain showed thermal stabilisation in the presence of divalent metal ions or endonuclease inhibitors, but no cleavage activity^27^. The structure of the RdRp has been predicted using pre-AlphaFold in silico methods, though the confidence of these models and their utility in antiviral development remained unclear^28^. Functional studies on the activity of the RdRp domain have identified D2517 as the key active site residue, likely coordinating divalent metal ions for NTP hydrolysis^29^. Polymerase activity was demonstrated using a single promoter RNA mimicking the 3′ end of the RNA template and a short complimentary primer and efficient inhibition of the activity by ribavirin or favipiravir^29^. While CCHFV-L is expected to contain a cap-binding domain in the C-terminal region this has not been identified.

In this study we sought to provide a characterisation of the molecular biology of the full-length CCHFV-L. We established robust expression and purification, allowing us to produce both wild type and several mutant constructs. Initial assessment of the endonuclease activity of the full-length protein revealed a highly active domain, with low metal ion specificity.

Guided by structure prediction and previous reports, residues were identified for mutagenesis to generate less active variants for downstream experiments. Single particle electron cryo-microscopy (cryoEM) was subsequently used to determine RNA free and 5′ promoter bound structure at high resolution. These models describe an RdRp domain of approximately 2200 amino acids in length and a unique arrangement of the 5′ promoter binding site. Functional characterisation reveals CCHFV-L to require the 5′ promoter bound for efficient production of RNA products. Collectively, our findings provide a detailed characterisation of a key enzyme from a potent human pathogen to support future antiviral development.

## Results

### Purification and solution characterisation of the CCHFV-L

**Fig. 1:**
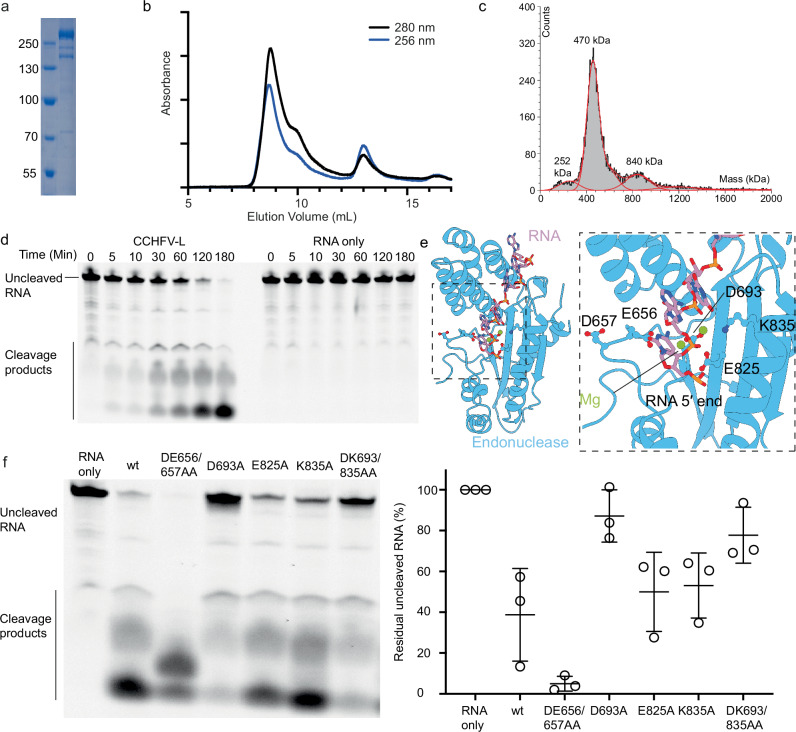
Full-length CCHFV-L contains an active endonuclease domain. **a** SDS-PAGE analysis of purified CCHFV-L. **b** Size exclusion chromatography on a S200 Increase 10/300 GL of CCHFV-L showing the absorbance traces at 256 (blue) and 280 (black) nm. **c** Mass photometry analysis of full length CCHFV-L. **d** Time course RNA degradation of a fluorescently labelled RNA template in the presence or absence of wild type (wt) CCHFV-L. **e** Alphafold 3 prediction of a CCHFV endonuclease domain (blue) in complex with magnesium (green) and a segment of RNA (pink). **f** Panel of the RNA degradation assay showing RNA products after incubation with CCHFV-L endonuclease proteins for 120 minutes. Quantitation’s of the uncleaved RNA band for three replicate experiments are shown for each mutant along with mean and standard deviations. The experiment was performed independently three times with similar results.

The gene encoding the full length *Orthonairovirus haemorrhagiae (*Crimean-Congo Haemorrhagic Fever Virus, CCHFV) CCHFV-L with an N-terminal OctaHis tag, a twinStrep, and a TEV-protease site was synthesised and cloned into the pFastbac vector for expression in SF9 insect cells. The protein was purified by affinity capture and size exclusion chromatography with SDS-PAGE analysis and absorbance showing a pure protein sample free from nucleic acid contamination (Fig. 1a/b). Analysis of the CCHFV-L solution state showed most particles had a mass matching that of monomeric CCHFV-L (Fig. 1c) (452.9 kDa from sequence). Additional species at 252 kDa and 840 kDa were also observed, potentially representing small populations of degraded proteins and oligomeric species, respectively.

### CCHFV-L Endonuclease activity

As previous studies have reported conflicting observations regarding the endonuclease activity of CCHFV-L, we first assessed the RNA cleavage activity of our CCHFV-L preparation. A 25 nt fluorescently labelled RNA substrate was incubated with CCHFV-L, and samples were collected at time points between 0 and 180 minutes (Fig. 1d). This experiment showed efficient endonuclease activity, highlighting substantial RNA degradation to short fragments within 120 minutes. No RNA degradation was observed when CCHFV-L was omitted.

Sequence alignments of nairovirus endonuclease domains have suggested residues that are potentially important for endonuclease activity^27^. To validate and expand the candidate list of important residues in the active site, we performed in silico modelling of the CCHFV endonuclease domain (residues 587-895) in complex with two magnesium ions and a 17 nt RNA fragment. The generated models showed high confidence in the overall domain architecture, confidently predicting the core structure but showing low confidence for two long insertion loops (Supplementary Fig. 1a). The residues that coordinate the active site magnesium ions and the ions themselves were predicted with high confidence (Supplementary Fig. 1b). The central portion of the RNA was predicted with moderate confidence, though this was sufficient to identify candidate residues for mutagenesis aimed at reducing endonuclease activity (Fig. 1e). Residues E656, D657, D693, E825, and K835 were mutated to alanine and then tested for endonuclease activity. Each mutant protein was assessed for cleavage after 120 minutes of incubation with the fluorescent RNA (Fig. 1f). The DE656/657AA double mutant substantially increased enzyme activity degrading the RNA to the detection limit of the technique. Given the location of these two residues in the active site, it may be that removal of these side chains enlarges the enzyme active site, enhancing the activity and producing a different RNA banding pattern. The wild type protein degraded approximately 65% of the input RNA as did the E825A and K835A single mutants. The DK693/835AA double mutant degraded approximately 25% of the RNA, while D693A alone degraded approximately 15%, substantially less than the wild type enzyme. Independent of band intensity, these mutants displayed a banding pattern similar to the wild type enzyme. In the predicted model both D693 and K835 directly coordinate the active site magnesium ion. Notably, the D693A mutant has been previously reported to inhibit mRNA production using a minigenome reporter system^26^. Considering levels of protein expression and reduction in endonuclease activity we chose the D693A mutant CCHFV-L for further structural and functional studies. Inhibition of endonuclease activity is important as the downstream experiments performed in this study require the addition of synthetic RNA which must remain undegraded^30^. While this work was under review a detailed structure and function analysis was published focused on the isolated endonuclease domain from two nairovirus species, supporting our finding that CCHFV contains a potent endonuclease activity^31^.

### Molecular architecture of the CCHFV-L core

To obtain structural information we prepared a sample of the full-length CCHFV-L. The resulting CCHFV-L structure exhibited a preferred orientation; however, through extensive and iterative classification and repicking we could recover minor populations of orthogonal particle views from the data (Supplementary Fig. 2a, b). The resulting reconstruction was resolved to a global resolution of 2.74 Å with regions of the map in the core of the L-protein extending to 2.4 Å (Supplementary Fig. 2c). A model was built and refined into the map using an in silico structure prediction as a starting point.

**Fig. 2:**
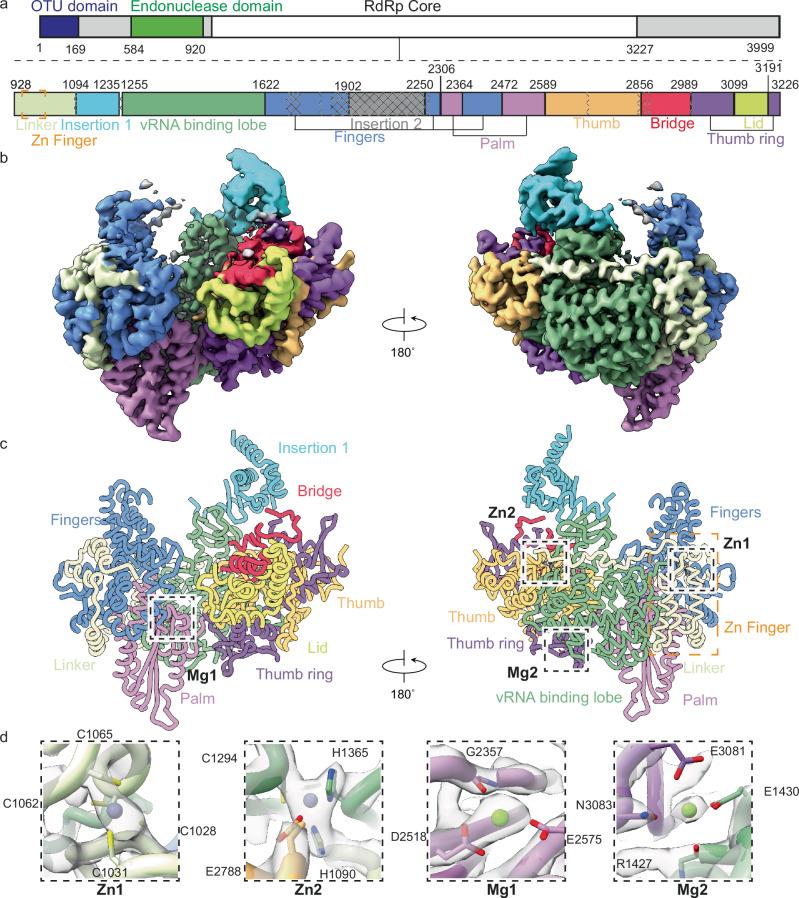
Molecular architecture of the CCHFV-L core. **a** Domain arrangement of the full-length CCHFV-L and the domains boundaries of the region of CCHFV-L we have determined. Hashed regions were not sufficiently ordered in our map to model. Annotations showing the Linker (light green), Zinc Finger (orange), Insertion 1 (cyan), vRNA binding lobe (green), Insertion 2 (grey), Fingers (dark blue), Palm (pink), Thumb (light orange), Bridge (red), Thumb ring (purple), and Lid (yellow/green) are shown. Colouring is conserved across all panels. **b** Density map and **c** molecular model of the CCHFV-L RdRp. Sites of metal coordination are highlighted. **d** Metal coordination sites are shown in zoom with interacting residues annotated.

The model encompasses residues 928-3226 and covers the endonuclease linker and the RdRp core of CCHFV-L (Fig. 2a, b). The region of the map likely corresponding to residues 1901-2263 appeared highly mobile. Although it could be observed at a low map threshold and despite extensive classification or flexible refinement, the quality was insufficient to build this region of the model. N-terminal regions containing the OTU and endonuclease domains or C-terminal domains were not observed.

From the N-terminus of our model we observe a 170 residue long endonuclease linker (residues 928-1093) that winds around approximately half of the RdRp (Fig. 2b, c). The linear path of the linker is interrupted by a three-helix zinc finger domain coordinating zinc ion 1 (Fig. 2d). This zinc ion is tetrahedrally coordinated by C1028/1031 at the N-terminus of one helix and C1062/1065 located at the C-terminus of the second helix. At the C-terminus of the linker a second zinc ion is coordinated by H1090, C1294 and H1365 from the vRNA binding lobe (VBL), and E2788 from the thumb domain (Fig. 2d). One zinc finger domain has been previously reported in the first 1325 residues of CCHFV-L^32^ and our data suggest it is likely those coordinating zinc ion 1. The structural role of these ions appears to be important in tethering the endonuclease linker tightly to the core of the RdRp. While structural metal ions are uncommon in polymerases of segmented RNA viruses^13^, they are frequently observed in the L-proteins of nonsegmented RNA viruses^33^. The residues involved in metal coordination also appear well conserved within nairoviruses (Supplementary Fig. 3).

Joining the endonuclease linker and the VBL is an additional folded domain, which we term insertion 1 (residues 1094-1235). A break in the density suggests that it is flexibly associated with these domains. Insertion 1 is formed by five α-helices and a three stranded β-sheet. We could not identify a structural homologue to the domain in other bunyavirus L-proteins. Insertion 1 occupies a similar position to that of the pyramid domain from Arenavirus L-proteins^13^, suggesting it may perform a comparable yet to be determined role. Similarly, this could be an expanded clamp region which has been observed in several bunyavirus L-proteins^17,18^. In Tomato Spotted Wilt Virus L-protein the clamp region comprises approximately 50 residues and undergoes an ordered/disordered rearrangement upon RNA binding^18^. We have not observed a similar transition in our models; however, they do not contain the 3′ genomic RNA which may be important for this rearrangement.

Residues 1255-1621 form the VBL that constitutes the binding site for the 5′ end of the viral genome. Residues 1235-1256 which link insertion 1 and the VBL are disordered. The VBL is followed by the tripartite fingers domain (residues 1622-1901, 2250-2306, 2365-2472) which is concatenated with the bipartite palm domain (residues 2307-2364, 2473-2589) (Fig. 2b, c). In many bunyavirus L-proteins both the fingers and the palm domains are bipartite, however, CCHFV-L has an extra insertion between residues 1902-2249 which was poorly resolved in the map. We have termed this insertion 2, and while it is not ordered in our maps, low threshold density suggests that it is flexibly located above the RdRp active site. Structural searches using FoldSeek^34^ revealed no detectable homology to enzymatic domains, suggesting a structural role.

Annotation of the RdRp core highlights the location of motifs A-G which are collectively critical for RNA and NTP binding (Supplementary Fig. 4a). Motif F, likely at residues 2272-2298, was only partially resolved. The RdRp active site contains the canonical double aspartate residues responsible for active site metal coordination, with residues 2517 and 2518 (Supplementary Fig. 4a). We observed a magnesium ion in the RdRp active site tightly coordinated by residues D2518, E2575, and the backbone carboxyl of G2357 (Fig. 2d). The Motif I, centred on residue K1620, is found in the hinge region between the fingers and VBL domains where it is predicted to interact with motif F.

Opposing the fingers domain are the thumb (residues 2589-2856), thumb ring (residues 2989-3099, 3191-3226), and lid (residues 3100-3190) domains. Another metal ion is coordinated by two residues from each the VBL and the thumb ring domain (Fig. 2d). The coordination by these four residues suggests an octahedral geometry, consistent with magnesium as the most likely metal. Residues 2857-2989 contains the bridge domain that links the thumb and thumb ring domains. In our model we observe three stretches of ordered residues, however approximately 60% of the residues in this region are not ordered. This region potentially contains the template exit loop and the priming loop.

### Binding of the 5′ genome to the CCHFV-L core

**Fig. 3:**
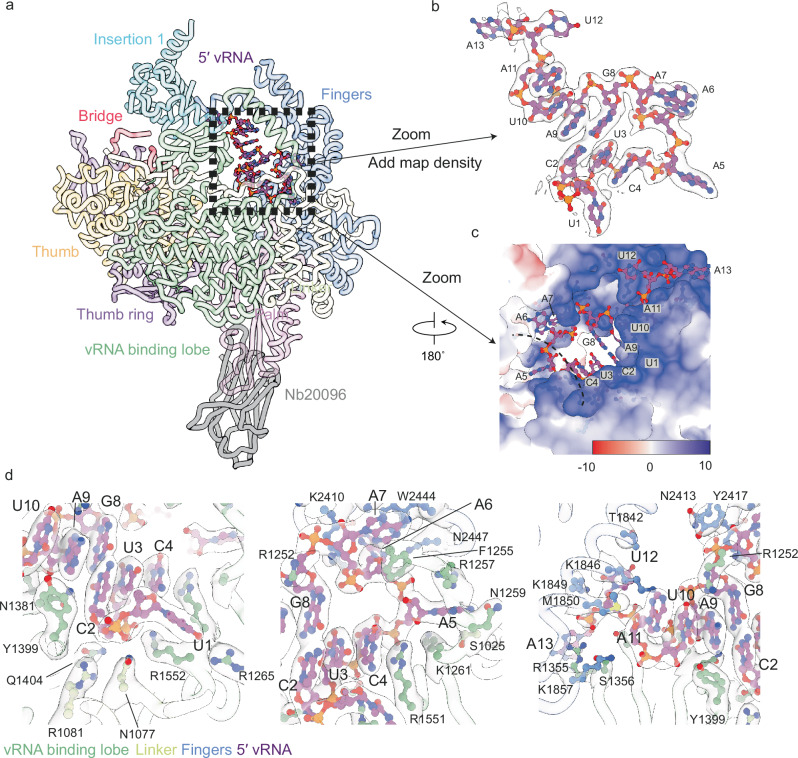
Coordination of the 5′ genome termini within the CCHFV-L. **a** The molecular model of the CCHFV-L to the 5′ genome termini in complex with Nb20096. Annotations showing the Linker (light green), Zinc Finger (orange), Insertion 1 (cyan), vRNA binding lobe (green), Fingers (dark blue), Palm (pink), Thumb (light orange), Bridge (red), Thumb ring (purple), Lid (yellow/green), and Nb20096 (grey) are shown. RNA is shown as atom representation with carbon (purple), phosphate (orange), oxygen (red), and nitrogen (blue). RNA and protein colour is conserved across panels. **b** Density map of the 5′ vRNA with the model fit (purple). Base numbers and identity are annotated. **c** Electrostatic potential surface representation of the RNA binding site highlighting strong electropositivity. The dashed line shows where residues have been removed to show the charge of the RNA binding site with more clarity. **d** Detailed description of the key residues involved in binding the 5′ hook into the CCHFV-L. Key residues have been annotated with bases showing in a larger font. The protein colouring follows the convention established in (**a**). The map contouring is shown for the RNA and residues at the same level to demonstrate modelling confidence.

We next sought to characterise the binding of the end of the 5′ genome to CCHFV-L. Purified CCHFV-L was mixed with a 15 times molar excess of 16mer synthetic RNA from the 5′ terminus (5′-UCU CAA AGA UAU AGC A-3′) and incubated on ice for 5 minutes. Immediately prior to preparing cryoEM grids a four times molar concentration of nanobody Nb20096 was added to help prevent potential issues with preferential orientation. From this dataset we were able to determine a complex describing the binding of the 5′ genome terminus, the CCHFV-L core, and the Nb20096 binding site to a global resolution of 2.31 Å (Fig. 3a, Supplementary Fig. 5a-c).

The 13 bases at the 5′ terminus of the genome were observed to occupy a site formed by the endonuclease linker, fingers domain, VBL, and insertion 1 domain (Fig. 3a-d, Supplementary Fig. 4b). The RNA-protein interface buries an interface of approximately 2100 Å^2^. Analysis of the electrostatic surface of the hook-binding site reveals a strongly electropositive region, as expected for coordinating the negatively charged RNA backbone (Fig. 3c). The 11 bases at the 5′ terminus form a tight hook structure, stabilised by a single Watson-Crick base pair between the cytosine at position 2 and the guanosine at position 8. The uridine in position 1 is flipped out form a base specific contact with R1265. Bases 2-4 form a three-base stack with the base of cytidine 2 and cytidine 4 forming π-stacking interaction with Y1399 and the planar head group of R1551, respectively (Fig. 3d, left). The arginine in position 1552 coordinates the phosphates from bases in position 3 and 4. The adenosine nucleotides in positions 5-7 have their bases flipped out with each base accommodated into a specific pocket in the CCHFV-L (Fig. 3d, middle). The phosphate of adenosine 5 is coordinated by R1551 while the sidechain of R1257 stacks against the base. The basic R1257 residue also coordinates the phosphate of adenosine 6 with the base occupying a hydrophobic pocket formed by L1623, I1629, W2444, and V2450. The base of adenosine 7 forms a stacking interaction with F1255 from the vRNA binding arch region and the phosphate from bases 7 and 8 are coordinated by K2410. Bases 8-11 form a four base stack that is stabilised by large regions of the VBL and fingers domain. These bases have fewer contacts with CCHFV-L than the earlier bases with key interactions from R1252, Y1399, and end of the base stack sandwiched by M1850 (Fig. 3d, right). Nucleotides 12 and 13 are outside of the hook structure and less well coordinated into CCHFV-L, making fewer and less base specific interactions. The density suggests K1846, K1849, K1857, and R1355 form a basic region for the phosphate into which the RNA backbone binds. The final three bases present in the RNA were not ordered likely due to limited interactions with the CCHFV-L.

**Fig. 4:**
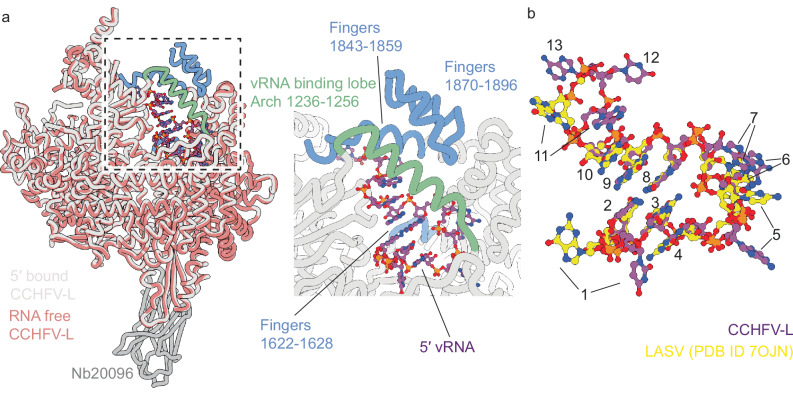
Effect of RNA binding and the 5′ hook structure. **a** Comparison of the RNA free (light red) and 5′ RNA bound structures (shades of grey). Regions which are ordered upon RNA binding are highlighted for the fingers (blue) or vRNA binding lobe (green). RNA is shown (purple). **b** Structural comparison of the 5′ RNA terminus between CCHFV (purple) and LASV (yellow) show strong similarity with conservation at bases 2-4 and 8-10 maintaining base pairing and stacking interactions.

Nb20096 binds to the palm domain, burying approximately 812 Å^2^ of surface area. The interface is maintained by residues from each of the three complementarity-determining regions (CDR) (Supplementary Fig. 4c, d). Notably, we observed a noncanonical interaction involving the residues N-terminal to CDR3, outside of the highly variable region, which contribute to a large area of the interface with the palm domain. Comparison of the palm domain in the presence or absence of Nb20096 did not show any differences (Fig. 4a).

Comparison of the 5′ vRNA bound and RNA free CCHFV-L models allow us to understand the changes caused by RNA binding. The 5′ promoter RNA is stabilised through the ordering of residues 1236-1256 in the VBL. These residues function similarly to the arch from the PA subunit of influenza virus polymerase^35^, Tomato Spotted Wilt Virus^18^ and Hantavirus L-proteins^20^. In addition, residues 1843-1859 and 1870-1896 from the fingers domain have also become ordered and pack on top of the arch and directly onto the RNA hook. Residues at the hinge region between the VBL and the fingers domain (residues 1622-1628) have also become well ordered, suggesting a rigidification of the CCHFV-L core (Fig. 4a). In neither of the 5′ promoter bound or RNA free models do we observe the full motif F structure, whereas previous studies have shown RNA binding to cause a rearrangement of this motif and an activation of the RdRp activity^17,18,22^. Similarly, neither model contained an ordered priming loop. Interestingly, previous activity studies of CCHFV-L have shown that addition of the 3′ template alone was a suitable template for efficient extension^36^, suggesting the 5′ promoter may not have the activating effect seen for other bunyaviruses.

Comparison of the 5′ promoter structure to that of other segmented RNA viruses shows the strongest homology to Lassa virus^13^. Similarities include the first base of the genome protruding out from the L-protein, the hook structure being maintained by a single C-G base pair between positions 2 and 8, and nucleotides 5-7 having extensive base-protein contacts (Fig. 4b). In contrast, other bunyavirus 5′ promoters have between 2-4 base pairs that stabilise the hook structure.

### Development of a radiolabelled NTP incorporation RdRp activity assay

**Fig. 5:**
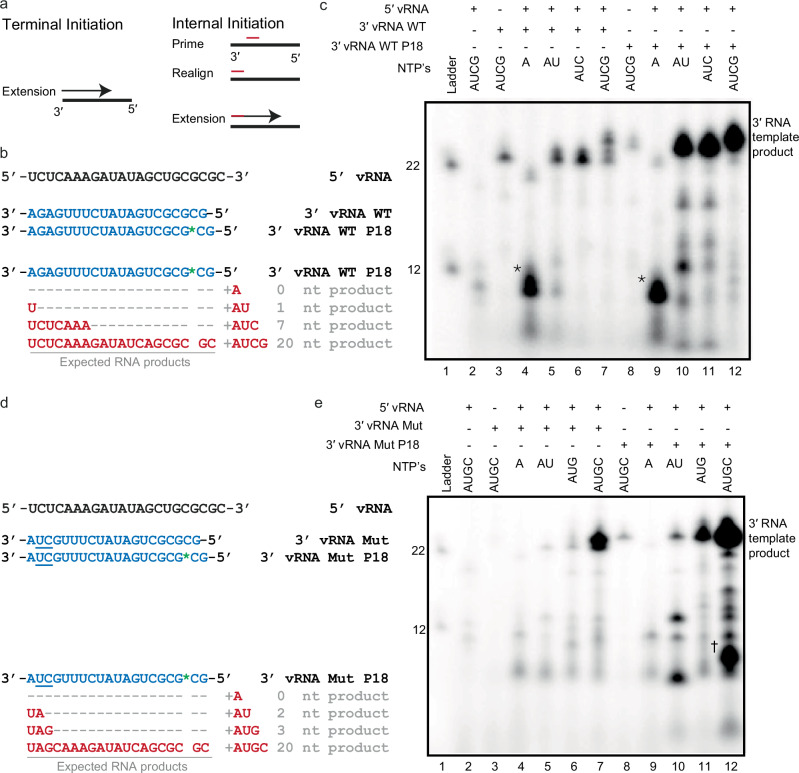
Unprimed radiolabelled extension assays. **a** Scheme showing the general mechanisms of terminal and internal initiation. RNA templates and expected products from terminal initiation with different NTP combinations are shown (**b**) and (**d**). The location of the Cy3 fluorophore is indicated as a green asterisk. Reaction products generated from (**c**) wild-type (WT) RNA or (**e**) mutant (Mut) RNA templates. * A polyA transcript of 10nt in length internally initiated opposite the UTP at position 5 in the template. † An abortive initiation product of length 5-8nt, unrelated to polyA transcripts noted in 5 C. Base exchanges in the mutant RNA template are underlined. Reaction conditions are detailed in the methods section. All assays use [α−32P]-ATP for labelling of RNA products. Assays were performed in duplicate with comparable results. 3′ vRNA are show in blue and expected product RNA and NTP in red.

We next sought to assess the activity of the RdRp within the full-length CCHFV-L protein to develop a screen for RdRp inhibitors. For this purpose, we produced a ^32^P-labeled radionucleotide extension assay. Previous work has shown that the polymerase efficiently extends an RNA mimic of the 3′ template^29^. As our structural analysis demonstrated that the 5′ terminus forms an integral component of the CCHFV-L core, we included both 5′ and 3′ termini in our assay templates. These RNAs contained the 13 bases from the 5′ terminus and the 15 bases from the 3′ terminus of the L segment of the genome (NC_005301.3). Previous analyses have shown that within the 5′ promoter, the nine terminal bases are highly conserved, as is a stretch from bases 17-21^37^. To mimic the interaction between the 5′ and 3′ RNA segments, we introduced a 5′-GCGCGC… sequence to the 3′ RNA template and the complement to the 5′ RNA template, producing a 21 nt long template. The 5′ and 3′ genome termini are complementary and thus may form a tight RNA dimer. To reduce the effect of this dimer on RNA synthesis, each RNA was denatured before being added sequentially to each reaction. Under unprimed reaction conditions, i.e. in the presence of free NTP, terminal or internal initiation may occur (Fig. 5a).

Using wild-type templates corresponding to the 5′ or 3′ termini of the genome (Fig. 5b), we observed that only limited extension products were produced (Fig. 5c, lanes 2 and 3). Addition of ATP yielded an abundant ~10 nt long product, likely produced from internal initiation from position 5 in the template RNA. Addition of UTP resulted in a product of approximately 22 nt, close to the expected full-length size (Fig. 5c, lanes 4 and 5). Further addition of CTP or GTP caused only minor changes in length and abundance of the observed products (Fig. 5c, lanes 6 and 7). The observation of a 22 nt product in the presence of only ATP and UTP suggests low fidelity of CCHFV-L where initiation can occur internally and noncomplementary bases are incorporated into product RNA. Synthesis of product RNA longer than the template is likely driven by cycles of template realignment early in initiation facilitated by repeating bases at the 3′ end of the template. Similar phenomena have been observed for related RNA virus polymerase under in vitro conditions^21,38^. The absence of viral nucleoprotein and a full-length viral RNA are likely significant contributing factors in these aberrant RNA products.

Using a mutant 3′ promoter (3′ vRNA Mut) where bases 2 and 3 are mutated from GA to UC (Fig. 5d) allowed us to control early stages of de novo initiation more precisely. In this system, only RNA products shorter that 3 nt would be produced unless all four NTP are present. Assays in the presence of a limited set of NTPs exhibited only low product abundance (Fig. 5e, lanes 4-6), while addition of all NTPs promoted generation of the ~22nt product (Fig. 5e, lane 7). Assays performed with wildtype or D693A mutant CCHFV-L showed similar activities in these assays, whilst D2517A/D2518A active site mutations inhibited RdRp activity (Supplementary Fig. 6d).

During assay development, we tested the activity of CCHFV-L on template RNAs containing fluorophores at several positions (Supplementary Fig. 6a). Incorporation of a Cy3 dye at base 18 of the wild-type 3′ template (3′ WT P18 vRNA) substantially enhanced product formation for both full-length and shorter products generated by the addition of ATP and UTP, or ATP, UTP and CTP (Fig. 5c). Fluorophores introduced at other positions inhibited or reduced activity of CCHFV-L (Supplementary Fig. 6b). In reactions using mutated 3′ templates with Cy3 incorporation at position 18 (3′ Mut P18 vRNA), the addition of ATP and UTP strengthened the production of a ~ 12 nt RNA product, whilst addition of the complete NTP set resulted in a ladder-like production of shorter transcripts alongside the strong full-length transcript (Fig. 5e, lanes 10 and 12). We speculate that the increase in activity observed due to incorporation of the dye at position 18 is related to a destabilisation of the predicted RNA duplex, perhaps enabling optimal positioning of the template RNA. Electromobility shift assays showed that the 3′ vRNA Mut P18 RNA did not anneal tightly to the 5′ vRNA (Supplementary Fig. 6c), suggesting weaker duplex formation between these two RNA.

### Development of a primed fluorescent extension assay

**Fig. 6:**
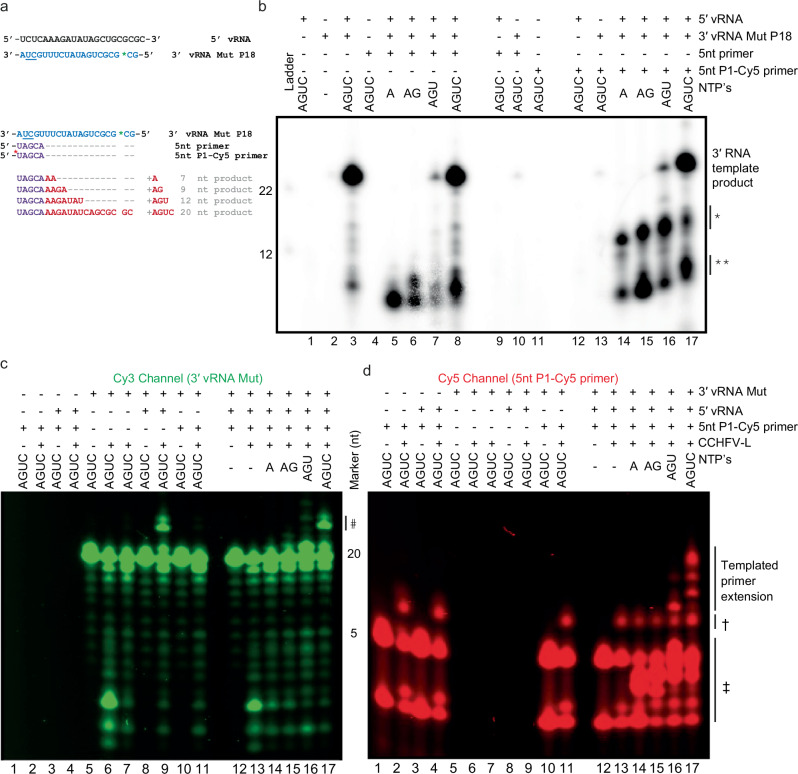
Primed radiolabelled and fluorescent extension assays. **a** RNA templates and expected products produced in the presence of NTP combinations with the addition of a 5 nt RNA primer. Location of fluorophores are indicated with a green (Cy3) or red (Cy5) asterisk. 3′ vRNA are show in blue, 5nt primer in purple, and expected product RNA and NTP in red. **b** Reaction products generated from a 3′ vRNA Mut P18 and 5′ vRNA templates with either the unlabelled 5 nt primer (lanes 4-10) or the 5 nt P1-Cy5 primer (lanes 11-18). * A >12nt reaction product appearing only in the presence of 3′ vRNA, 5′ vRNA, and P1-Cy5 primer is likely due to partial extension of the P1-Cy5 primer with ATP alone. ** Abortive initiation products formed from the extension of either the unlabelled or P1-Cy5 primer by CCHFV-L. The short abortive products are extended on addition of GTP, or reduce on addition of UTP, when longer products are formed. **c**, **d** Fluorescent reaction products generated from the Mut P18 vRNA template together with the 5′ vRNA template and P1-Cy5 primer, split into Cy3 and Cy5 fluorescence channels. #A Cy3-labelled fluorescent extension product in the presence of Mut P18 vRNA, CCHFV-L and 5′ vRNA likely indicates nontemplated extension of the 3′ Mut P18 vRNA. † A Cy5-labelled band over 5 nt in length is the product of CCHFV-L and P1-Cy5 primer in the absence of NTPs, indicating an interaction between the L-protein and P1-Cy5 primer independent of RdRp activity. ‡ Cy5-labelled fluorescent bands shorter than 5 nt are likely derived from extension using a degraded 5 nt P1-Cy5 primer. Assays were performed in duplicates with comparable results.

Our unprimed assays suggested strong polymerase activity; however, we suspected that the early stages of initiation may be prone to nontemplated extension. To promote template-dependent initiation and identify whether primer-driven initiation was possible with the CCHFV-L protein, we developed a primer-dependent assay using a 5 nt RNA primer (Fig. 6a). We synthesised RNA primers without a label and with a Cy5 fluorophore attached to the 5′ of the U1 nucleotide (P1-Cy5 primer).

Reactions including only the 3′ template and primer resulted in very reduced product formation (Fig. 6b, lanes 10 and 13). Further inclusion of the 5′ strand enhanced the production of the 22 nt product (Fig. 6b, lanes 8 and 17), suggesting a strong activating role for the 5′ RNA template. Previous studies have shown extension using just a 5 nt primer and 3′ template RNA^29^, however our structural data suggests that the 5′ end is also required, a concept which is strongly supported by our functional data.

Withholding nucleotides in these assays limited the extension of products when reactions were initiated with the unlabelled primer (Fig. 6b, lanes 5–8). On addition of ATP we observed a dominant short reaction product at ~7 nt in length. We noted another stronger extension product at ~9 nt on further addition of UTP, whilst further addition of GTP produced a weak full-length reaction product. Using all NTPs, we observed a strong full-length product with additional short extension products being visible.

To differentiate between de novo extension products and primed extension products, we developed a fluorescence extension assay using the P1-Cy5 primer. Upon sequential addition of NTPs, we observed the production of increasingly longer products similar to the reactions using unlabelled 5 nt primer (Fig. 6b, lanes 14–17). In addition, we observed an abundant intermediate product at the length of ~12 nt. We believe this product is directly related to the fluorescent primer, as the presence of the fluorophore is the only difference between the reactions. The molecular identity of the product is unclear, though it may be related to synthesis issues producing a degraded product.

We next replicated the conditions of the radiolabelled extension assay, however using the fluorescently labelled 5 nt primer to observe RNA products (Fig. 6c, d). We could clearly observe the extension of the primer in ATP/UTP/GTP conditions, producing an extension product of ~13 nt. Addition of CTP produced a strong fluorescent product at ~20 nt in length, indicating a full-length product accompanied by intermediate products. Collectively our fluorescent assays demonstrate the utility of this technology for analysing RNA extension by CCHFV-L and as a potential approach for reformatting into a high throughput antiviral screening system.

### Screening of nucleotide triphosphate analogues

**Fig. 7:**
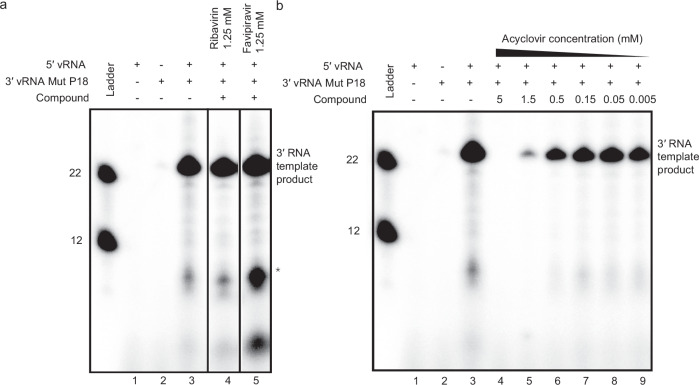
Screening of antiviral nucleotide triphosphate analogues. **a** Reaction products generated from 3′ vRNA Mut P18 and 5′ vRNA templates in the presence of Ribavirin-TP and Favipiravir-TP at 1.25 mM concentration. The assay was run in duplicate, producing comparable results. *A truncated extension product generated by the incorporation of Favipiravir at positions 5-9 in the RNA transcript, resulting in the stalling and abortion of transcript extension. **b** Reaction products generated from 3′ vRNA Mut P18 and 5′ vRNA templates in the presence of Acyclovir-TP. Acyclovir-TP concentration was titrated from 5 mM to 5 µM. All lanes contain DMSO at 25% v/v. Reaction conditions are detailed in the methods section. Assays were performed four times with comparable results.

We next turned our attention to inhibiting the RdRp domain of CCHFV-L using commercial nucleotide analogues. Ribavirin-TP and Favipiravir-TP, guanosine and purine nucleotide analogues respectively, were selected as broad-spectrum antivirals with demonstrated activity against RNA virus polymerases^39,40^. Each compound was screened for activity in our ^32^P radiolabelled extension assay at a final concentration of 1.25 mM (Fig. 7a, lanes 4 and 5). Addition of Ribavirin-TP and Favipiravir-TP at high concentrations did not reduce the full-length product. Previous work has shown that incorporation of Ribavirin-TP and Favipiravir-TP are incorporated into product RNA at a 90-700 fold lower rate compared to purine nucleotides^29^. We note that, Favipiravir-TP enhanced the formation of a truncated product at less than 12 nt in length. The titration of lower compound concentrations had no effect (Supplementary Fig. 6e). Favipiravir-TP appears to have a dual mode of action resulting in partial chain termination where consecutive molecules are incorporated into the product^40^ and can also induce mutation in subsequent rounds of genome copying^41^. In the context of our assay a series of pyrimidines at positions 5-9 would likely allow for this consecutive incorporation and stalling, producing the abortive product. Ribavirin acts as a mutagenic base in subsequent rounds of replication, templating incorporation of cytidine or uridine with equal efficiency^39^. We are unlikely to observe this phenotype in our assay.

Our results contrast with some previous literature which observed chain termination for both analogues in a system using only the 3′ RNA strand and a 4 nucleotide primer^29^. It may be that the absence of the 5′ promoter, using a primer as opposed to our de novo primed reactions, differences in the compound to NTP ratio, or template sequence led to this discrepancy. In a biological context ribavirin treatment was not shown to increase the rate of mutations^42^ while the utility of either compound in a clinical context is unclear^7^.

We next screened Acyclovir-TP, a chain terminating acyclic purine nucleoside analogue used to specifically target the DNA polymerase of herpes viruses since the 1980’s^43^. We titrated Acyclovir-TP into the radionucleotide extension assays from 5 mM to 5 μM concentrations (Fig. 7b) showed complete inhibition of full-length product formation at the highest concentration (Fig. 7b, lane 4), while at lower concentrations we only noted a partial reduction.

## Discussion

All bunyavirus L-proteins are thought to contain certain key features including an endonuclease domain, an RdRp domain, and a cap-binding domain. For most bunyaviruses with animal hosts the resulting L-protein is approximately 250 kDa. Here we have provided the characterisation of the function for two of the enzymatic domains and two high resolution structures describing the core RdRp of CCHFV-L.

The L-protein RdRp core we describe here contains approximately 2130 residues, of which we observe 1505 in our RNA bound model, which is much larger than the RdRp from previous structurally characterised bunyaviruses including arenaviruses ( ~ 1550 residues^14^), hantaviruses ( ~ 1340 residues^21^), peribunyaviruses (1470^16^), or phenuivirises (1340 residues^11^) (Supplementary Fig. 7). This extra mass is largely accounted for by two unexpected structural domains that we have termed insertion 1 and insertion 2, which are approximately 140 and 350 residues, respectively. Comparison to structures of other bunyavirus RdRps shows that the TSWV-L^18^ and LASV-L^14^ each have insertions into the RdRp, in a similar location as the insertion 1 in CCHFV-L (Supplementary Fig. 7). In TSWV-L the clamp domain is ordered upon addition of vRNA, while in our models it is similarly arranged in the presence or absence of RNA. We observe no structural homology between the insertion 1 and clamp domains, it appears only the insertion into the RdRp is conserved. The insertion 2 domain in CCHFV-L appears conserved within nairoviruses (Supplementary Fig. 3) but is not apparent in other bunyavirus families. In our models it is poorly ordered, and we were unable to determine its functional role. Comparison of the endonuclease linker from other bunyavirus L-proteins demonstrates that CCHFV-L is distinct in containing a zinc finger domain (Supplementary Fig. 7), the functional importance of this is yet to be understood.

From the combined ^32^P radiolabelled and fluorescence extension assays, we can draw several conclusions regarding CCHFV-L RNA product initiation and extension. Our functional data consistently demonstrated that the activity of CCHFV-L is potently activated by the addition of the 5′ genome RNA, generating a strong increase in the replication activity of the protein compared to 3′ template alone (Fig. 5c, lanes 3 and 7, and 8 and 12, Fig. 5e, lanes 3 and 7, and 8 and 12). This finding begins to reveal the requirements of template-dependent initiation, and provides a platform for further investigations into the CCHFV-L transcription and replication initiation cycles. The more distantly related influenza virus polymerase have been demonstrated to show a strong increase in activity with the addition of promoter RNA^44^. Studies on the more closely related arenavirus L-proteins showed that the addition of the 5′ vRNA promoter increased the activity of the LASV-L in de novo replication but reduced activity in cap-primed assays^14^. Given the comparable promoter structure of Arenaviruses and CCHFV-L (Fig. 4b), it may be that they function in a similar manner in CCHFV.

We demonstrate that CCHFV-L can incorporate nontemplated nucleotides into the extending product in the absence of a complete NTP set (Fig. 5c lanes 4–6 and 9–11, and Fig. 5e lanes 4–6 and 9–11). This behaviour was not unexpected considering the ability of other segmented negative strand viral polymerases to also produce nontemplated products^21,38^; we expect that many of these products are the result of our experimental setup.

The unexpected product’s may also arise from slippage of the 3′ end of the vRNA within the RdRp active site. While one of several bases near the 3′ end of the vRNA may transiently occupy the active site, extension is favoured when the positioned base matches NTP’s present in the cognate pool. Depending on which NTP are available, extension of the RNA may then proceed. Because initiation is considered the rate-limiting step, correct base pairing at this stage is more critical than during later stages of elongation.

Under native RNP conditions the CCHFV-L would typically interact with a whole genome rather than the limited RNA sequence here and will likely be affected by the absence of the nucleoprotein, which in negative strand viruses impacts RNA synthesis^45^. Finally, we have also shown that CCHFV-L is capable of de novo and primed template-initiated RNA synthesis (Fig. 5c and e, lanes 7 and 12, Fig. 6c and d, lane 17). This primer-initiated synthesis corroborates previous work demonstrating the utility of CCHFV-L to extend a short RNA primer^29^.

Our antiviral screening experiments unexpectedly identified Acyclovir-TP as an inhibitor of the CCHFV-L (Fig. 7b). Typically, Acyclovir is used against Herpes viruses where in the tri-phosphorylated form it acts a potent chain terminator, inhibiting the function of the viral polymerase. We are yet to observe active production of RNA by CCHFV-L with cryoEM, but future studies will aim to reveal the molecular details of this inhibition. Potentially this will open up novel approaches for antiviral design.

Collectively, we have provided a structural and functional description of a pathogenic nairovirus L-protein. These findings provide a fundamental advance in our understanding of CCHFV processes and provide new avenues for antiviral discovery.

## Methods

### Protein cloning and purification

The viral L-protein gene from *Orthonairovirus haemorrhagiae* (NCBI Reference Sequence: YP_325663.1) was synthesised (SynBio) with N-terminal twin-strep and octa-His tags followed by a TEV protease cleavage site in a pFastBac vector. Genes encoding mutations were generated using standard cloning techniques. Baculoviruses for all viruses were generated according to standard protocols in sf9 insect cells (Sourced from the STRUBI cell bank, University of Oxford) maintained in serum-free Sf-900 media (Gibco). For large scale protein expression cells were infected with virus and left for 72 hours. After harvesting, the cell pellet this was resuspended in a buffer containing 50 mM HEPES, pH 7.5, 500 mM NaCl, 0.05% (w/v) n-Octyl beta-D-thioglucopyranoside, 2 mM dithiothreitol, 10% (v/v) glycerol, one protease inhibitor tablet (Sigma), 5 mg RNAse, and 1.2 mL of BioLock (IBA). After 30 minutes cells were further lysed by sonication, clarified with centrifugation and the supernatant incubated with Strep-Tactin Superflow high capacity (IBA) resin for 2 hours. Resin was washed with 100 column volumes of buffer comprising 50 mM HEPES, pH 7.5, 500 mM NaCl, 0.05% (w/v) n-Octyl beta-D-thioglucopyranoside, 2 mM dithiothreitol, and 5% (v/v) glycerol. Prior to elution a final wash with 20 column volumes of 50 mM HEPES, pH 7.5, 500 mM NaCl, 1 mM dithiothreitol, and 5% (v/v) glycerol. CCHFV-L was then eluted with 10 column volumes 20 mM HEPES, pH7.6, 500 mM NaCl, 1 mM dithiothreitol, 5% (v/v) glycerol, and 50 mM Biotin. As required CCHFV-L was passed over a Superdex 200 Increase 10/300 column into a buffer containing 20 mM HEPES, pH7.6, 500 mM NaCl, 1 mM dithiothreitol, 5% (v/v) glycerol. The sample was then concentrated and snap frozen in liquid nitrogen prior to storage at −70 °C.

### Nanobody generation, selection, expression, and purification

Llama immunizations, phage display library construction and nanobody purifications were performed according to earlier described protocols^46^. Briefly, one llama (Lama glama) was immunized with 650 µg of purified recombinant CCHFV-L over a period of 6 weeks and a nanobody library in pMESy4 of 2.4 ×10^8^ independent clones was established. CCHFV‑L–specific phages were retrieved by biopanning on CCHFV‑L–coated wells. After elution with trypsin, AEBSF was added to neutralize any residual trypsin activity. The resulting eluate, containing the released phages, was then used to infect freshly grown TG1 cells. Following two rounds of selection, individual colonies were screened by ELISA for the expression of CCHFV‑L–specific nanobodies. In total, 31 distinct nanobody families were identified according to their CDR3 regions.

For expression and purification, a plasmid containing the nanobody-20096 (Nb20096) gene with a C-terminal His tag, was transformed into chemically competent Escherichia coli WK6 cells. Cells were then grown in LB media supplemented with 0.1% glucose (w/v), 1 mM MgCl_2_, and 100 mg/mL ampicillin to a optical density of 0.7 prior to overnight induction with 1 mM isopropylthiogalactoside at 28 °C. After centrifugation to collect the cells, nanobodies were released from the periplasm by osmotic shock. The supernatant was then clarified by centrifugation, bound to NiNTA resin (Qiagen), before elution with 500 mM imidazole. Once concentrated the elution was then applied to a Superdex S75 Increase 10/300 GL column equilibrated in 20 mM HEPES, pH7.6, and 150 mM NaCl. Fractions which contained the Nb20096 were again concentrated and stored at −20 °C. Details of Nb20096 have been deposited in the NanoSaurus database.

### Mass photometry

Experiments were performed on a TwoMP mass photometer (Refeyn). Prior to conducting experiments, a calibration with three standards (thyroglobulin, ovalbumin, and aldolase) were each diluted in buffer containing 20 mM, HEPES, pH 7.5, 500 mM NaCl, 0.5 mM dithiothreitol, and 5% (v/v) glycerol. For CCHFV-L analysis the protein was diluted to a final concentration of 0.01 mg/ml in the same buffer. Analysis was performed using the Dis-coverMP software.

### Endonuclease cleavages assays

A Fluorescein dye labelled 25-mer ssRNA substrate (5′- UAGUAGUAUGCUCCGCAGGAACAAA-3′), was chemically synthesised (Integrated DNA Technologies). This RNA is unrelated to the nairoviruses and was designed in a previous study where it was shown to be a good substrate for endonuclease domains. Endonuclease activity assays were performed by incubating 2 μg CCHFV-L with 2.5 μM of 25-mer ssRNA and 2.5 mM of MgCl_2_. The reactions were incubated at 30 °C at time intervals ranging from 0-180 minutes. At each time point, the sample tube was removed and reaction stopped by addition of RNA loading buffer to a final concentration of 45% formamide and 5 mM Ethylenediaminetetraacetic acid (EDTA) prior to heating samples to 95 °C for 3 minutes. Reaction products were resolved by 7 M Urea 20% polyacrylamide Tris-borate-EDTA (TBE) gel electrophoresis (PAGE) in 0.5X TBE buffer. Fluorescent signal was detected using Chemidoc MP (Biorad). Uncropped images are provided in the source data file.

### Cryo-EM sample preparation

Building from our early work demonstrating the CCHFV-L contained an active endonuclease domain, our cryoEM experiments were prepared using the D693A mutant. All grids were prepared using a Vitrobot mark IV (FEI) at 100% humidity. UltrAuFoil (Quantifoil) (R2/2 on a 200 mesh or R1.2/1.3 300 mesh) grids were glow discharged, before a volume of 3.5 μL sample was applied and blotted for 5.5 seconds before vitrification in liquid ethane.

The RNA free CCHFV-L structure was prepared by directly diluting frozen protein stock to a final concentration of 0.8 mg/mL with 20 mM HEPES, pH7.6, 500 mM NaCl, and 5% (v/v) glycerol.

The CCHFV-L-Nb20096-5′RNA sample contained CCHFV-L at a final concentration of 0.6 mg/mL ( ~ 1.3 μM), Nb20096 at a concentration of 0.06 mg/mL ( ~ 5 μM), and 20 μM 5′ RNA (5′-UCU CAA AGA UAU AGC A-3′). RNA was incubated with CCHFV-L for 5 minutes prior to the addition of Nb20096 and the grids prepared immediately.

### Cryo-EM image collection

Cryo-EM data for the RNA free CCHFV-L were collected at the Oxford Particle Imaging Centre, on a 300 kV G3i Titan Krios microscope (Thermo Fisher Scientific) equipped with a SelectrisX energy filter and Falcon IV direct electron detector. Data were collected automatically in EPU 3.4, movies were recorded in EER format with a total dose of ~50 e-/Å^2^ and a calibrated pixel size of 0.932 Å/pix.

The CCHFV-L-Nb20096-5′RNA was collected at the EMBL Imaging Centre in Heidelberg, on a 300 kV G4 Titan Krios microscope (Thermo Fisher Scientific) equipped with a SelectrisX energy filter and Falcon IV direct electron detector using SerialEM version 4.2.0beta^47^. Data were collected in EER format with a total dose of ~50 e-/Å^2^ and a pixel size of 0.73 Å/pix. As preferential orientation had been observed in screening samples 7445 movies were collected at a 0° before the stage was tilted to 30° and a further 5784 movies collected.

### Cryo-EM data processing

Data were processed using cryoSPARC V4.7.1 using the same initial process^48^. EER format movies were fractionated in 40 frames without applying an up-sampling factor. Patch motion correction and patch CTF-estimation were performed with default setting, movies with poor statistics were removed. To generate a first round of candidate particles for processing blob picker was used followed by 2D classification. These initial templates were then used for template-based picking. These two particle sets were merged, duplicates removed, 2D classified, and the classes containing high resolution information used to train a Topaz model^49^. Particles from the different picking approaches were then pooled, duplicates removed, and a first round of ab-initio models generated and subsequently refined using heterogenous refinement. Particles which classified into the high-resolution heterogenous classes were then used to retrain a Topaz model and the process was iterated twice. The final high-resolution heterogenous class was then further refined using nonuniform refinement (with per particle CTF refinement) followed by a local refinement. This map was then considered the consensus refinement. For the RNA free dataset this was where processing stopped. For the RNA bound dataset we performed further 3D classification to remove particles which did not have RNA bound before a final round of local refinement.

### Structure determination and model refinement

To generate an initial protein model the AlphaFold 3 server^50^ was used. The map and model were imported into UCSF ChimeraX^51^, globally positioned in the map and large regions not observed in the model removed. Cycles of manual refinement and rebuilding in Coot^52^ and real space refinement in PHENIX^53^ were used to generate a final model. For the 5′ RNA model, the RNA was built de novo in coot. Final model geometry and map-to-model comparison was preformed using PHENIX Molprobity. Once the model had been built for the RNA bound complex this was used as a template for generating the RNA free model with cycles of refinement in coot and PHENIX. All map and model statistics are described in Supplementary Table 1. Structural analysis and figures were prepared using UCSF ChimeraX^51^.

Endonuclease domains were predicted in complex with a 17 nucleotide RNA (5′-GCCGCCGCCAUGAAUAA-3′). Several RNA sequences including those comprised of a single base, however many of these demonstrated self-annealing, confusing analysis of the binding site. The above sequence was chosen as it contains a series of bases which are not complimentary and thus were predicted as a single stranded RNA sequence.

### In vitro activity assays

Wild-type and D693A CCHFV L proteins were used in radiolabelled and fluorescent extension assays. In the radiolabelled assay, CCHFV L protein at a final concentration of 1 μM was initially diluted in a reaction buffer containing 20 mM HEPES pH 7.5, 100 mM NaCl, 1 mM DTT, 10% glycerol, 5 mM MgCl_2_ and 5 mM MnCl_2_. The diluted L protein was added to a reaction mixture with final concentrations of 3′ template, 5′ template, and 5nt primer RNA at 250 nM, RNAsin Plus (Promega) at 1U/µl, GTP, CTP and UTP at 125 μM, ATP at 250 nM, and [α−32P]-ATP (Revvity) at 0.2 µl/reaction, added to a final volume of 3 μl. Reactions progressed at 30 °C for 1 hour before an identical volume of Formamide dye (90% formamide, 10 mM EDTA pH 8) was added, and samples were heated to 80 °C for 5 minutes. Samples were loaded onto 6 M urea, 20% polyacrylamide denaturing gel (dimensions 16.5 × 28 cm (w x h), C.B.S. Scientific) and run at 450 V for 4 hours to resolve RNA products. Radioactive extension signal was quantified through phosphorimaging screen exposure and subsequent scanning (Typhoon FLA 9500). Acyclovir-TP, Favipiravir-TP, and Ribavirin-TP were kind gifts from Ervin Fodor in the Sir William Dunn School of Pathology, University of Oxford. Compounds were dissolved to a final concentration of four times the concentration used in the assay in 100% v/v DMSO before being added to the reaction at the final concentration.

Fluorescent RNAs except for the P1-labelled 5 nt primer (Dharmacon) were ordered from Integrated DNA technologies (IDT). Cy3, and Cy5 fluorophores were conjugated to the oligonucleotides through phosphoramidite chemistry for 5′, 3′, and internally positioned fluorophores.

The fluorescent extension assay proceeded as described for the radiolabelled extension assay, except that unlabelled ATP instead was added at a final concentration of 125 μM. Fluorescent signal was visualized using the Chemidoc imaging system (Bio-Rad), with separate Cy3 and Cy5 channels used to discriminate between the two fluorophores.

### RNA binding assays

3′ vRNA Cy3 labelled RNAs WT P4, Mut P4, Mut P14, WT P18, Mut P18 and WT P20 were diluted with unlabelled 5′ vRNA (21nt in length) to a final concentration of 300 nM each in hybridization buffer (20 mM Tris-HCl pH 8, 500 mM NaCl, 1 mM EDTA pH 8). Mixtures were incubated on a thermocycler with a single cycle temperature gradient from 95 °C to 4 °C. This gradient decreased in 2-minute, 2.5 °C intervals from 95 °C to 67.5 °C, followed by a drop to 65 °C for 5 min, 45 °C for 10 min, 25 °C for 5 min, and then held at 4 °C. Following annealing, the 300 nM RNA were mixed 1:1 with native loading solution (62.5 mM Tris-HCl, pH 6.8, 40% glycerol). Samples were then loaded onto a 4%–15% Bio-Rad Mini-PROTEAN TGX gels and run in SDS-free tris-glycine running buffer. Gels were visualized using a Bio- Rad Chemidoc imaging system in the Cy3 and Cy5 emission ranges.

### Reporting summary

Further information on research design is available in the Nature Portfolio Reporting Summary linked to this article.

## Supplementary information

Supplementary Information.

Reporting summary

Transparent Peer Review file

## Source data

Source data

## Data Availability

CryoEM maps and models generated in this study have been uploaded to the PDB and EMDB. The accession codes for the RNA Free model are 9T0F and EMD-55400. For the RNA bound model they are 9T0E and EMD-55399. Accession codes for models determined in previous work are for LASV [10.2210/pdb7ojn/pdb], TSWV [10.2210/pdb8ki7/pdb], SFTSV [10.2210/pdb8asb/pdb], HTNV [10.2210/pdb8cv4/pdb], and LCAV [10.2210/pdb6z8k/pdb]. Source data are provided as a source data file. Source data are provided with this paper.
