# Supplementary material for: Structural and functional characterisation of the Crimean-Congo haemorrhagic fever virus RNA dependent RNA polymerase
Source: Nat Commun. 2026 Jun 9;17:7338. doi: 10.1038/s41467-026-74113-w (PMC13402591; doi:10.1038/s41467-026-74113-w)
Supplement: Supplementary file 1 — Supplementary Information. [file 41467_2026_74113_MOESM1_ESM.pdf]

Structural and functional characterisation of the Crimean-Congo Haemorrhagic Fever Virus RNA Dependent RNA Polymerase

Adrian Deng[1*], Rory Cunnison[2*], Loïc Carrique[3*], Franziska Günl[4], Els Paddon[5,6], Jan Staeyart[5,6], David Nguyen Duc[1], Jonathan M. Grimes[3], Nicole Robb[2], and Jeremy R. Keown[1]

1 School of Life Sciences, University of Warwick, Coventry, United Kingdom
2 Warwick Medical School, University of Warwick, Coventry, United Kingdom
3 Division of Structural Biology, Centre for Human Genetics, University of Oxford, Oxford, United Kingdom
4 Sir William Dunn School of Pathology, University of Oxford, Oxford, United Kingdom
5 Structural Biology Brussels, Vrije Universiteit Brussel, VUB, Brussels, Belgium
6 VIB-VUB Center for Structural Biology, VIB, Brussels, Belgium

*These authors contributed equally towards the project: Adrian Deng, Rory Cunnison, and Loïc Carrique.

email: jeremy.keown@warwick.ac.uk

Supplementary information. Table 1 and Figures 1-7.

		CCHFV-L + 5'vRNA + NB20096<br>EMD-55399<br>9T0E	CCHFV-L<br>EMD-55400<br>9T0F
**Data collection**			
Microscope		Titan Krios (EMBL)	Titan Krios(OPIC)
Voltage (kV)		300	300
Detector		Falcon 4i - SelectrisX	Falcon 4i - SelectrisX
Recording mode		eer	eer
Magnification		165,000	130,000
Movie/micrograph pixel size (Å)		0.73	0.932
Dose rate (e-/px/sec)		11.1	9.27
Number of frames per movie		40	40
Movie exposure time (s)		2.4	4.9
Total dose (e-/Å^2)		50	50
Defocus range (um)		1.4 to 2.6	1.4 to 2.6
**EM data processing**			
Number of movies/micrographs		13,240	14,536
Box size (px)		380	300
Particle number (After initial 3D Classes)		252K	350K
Particle number (used in final map)		52k	268K
Symmetry		C1	C1
Map resolution (Å, FSC 0.143)		2.31	2.74
Local resolution range (Å, FSC 0.5)		2.05 - 30	2.47 - 30
Map sharpening B-factor (Å^2)		52	108
**Model Building and Validation**			
Initial model used		AlphaFold3 prediction	AlphaFold3 prediction
Model composition			
Non-hydrogen protein atoms		29391	25817
Protein residues		1789	1604
Nucleotides		13	/
Waters		293	/
Ligands	Zn	2	2
	Mg	2	2
B factors (Å^2) - min/max/mean			
Protein		29.61/129.39/60.66	32.08/149.98/67.05
Nucleotide		48.98/90.87/62.92	/
Waters		30.49/67.39/43.11	/
Ligands		36.04/71.88/51.62	37.17/93/74/63.16
RMSD from ideal			
Bond length (Å)		0.005	0.003
Bond angles (°)		0.572	0.51
Validation			
Molprobity score		1.45	1.39
Clashscore		4.64	5.38
Rotamers outliers (%)		1.17	0
FSC (0.5) model-vs-map		2.5	3
CC model-vs-map (masked)		0.83	0.71
Ramachandran plot			
Favored (%)		97.1	97.53
Allowed (%)		2.78	2.41
Outliers (%)		0.11	0.6

Supplementary Table 1. Cryo-EM collection and refinement parameters.

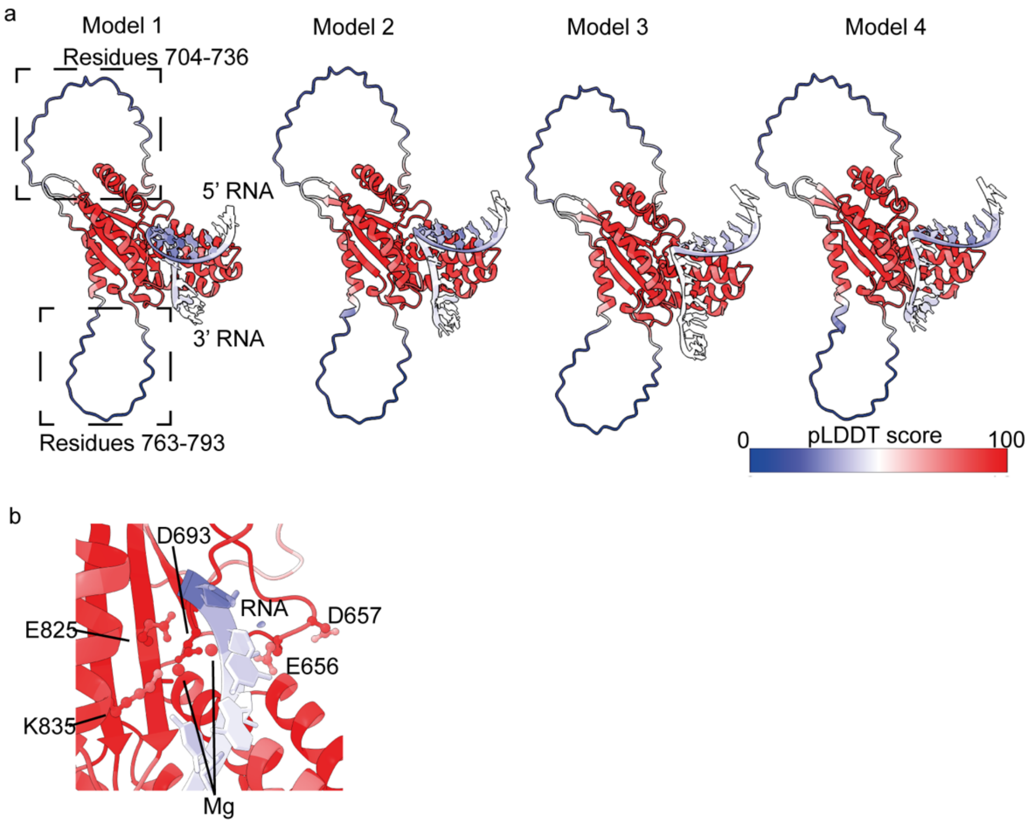

Supplementary figure 1. CCHFV endonuclease predictions bound to RNA and magnesium. a) Four models of the endonuclease domain have been predicted using the Alphafold Server. Models are coloured according to the pLDDT score. Residues of the endonuclease which are predicted with poor accuracy are shown in boxes. b) Zoom of the model 1 endonuclease domain active site showing RNA (sticks), magnesium (sphere), and potentially important residues (sticks). Colouring as per the pLDDT score shown in panel a).

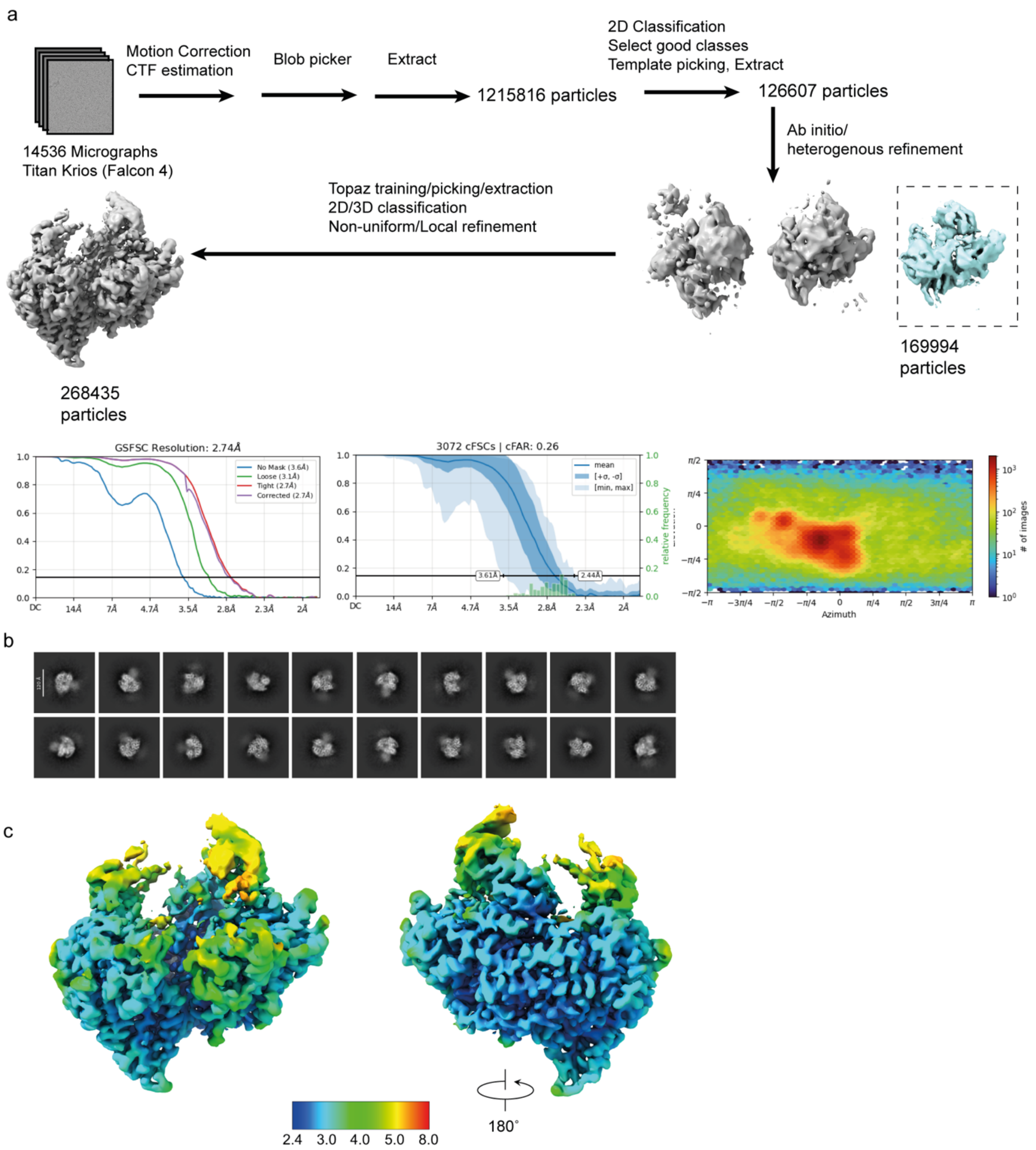

Supplementary figure 2. Cryo-EM processing workflow for RNA free CCHFV-L.
a) Processing scheme for the RNA free CCHFV-L. b) 2D classes of CCHFV-L. c) Resolution range of the resulting map.

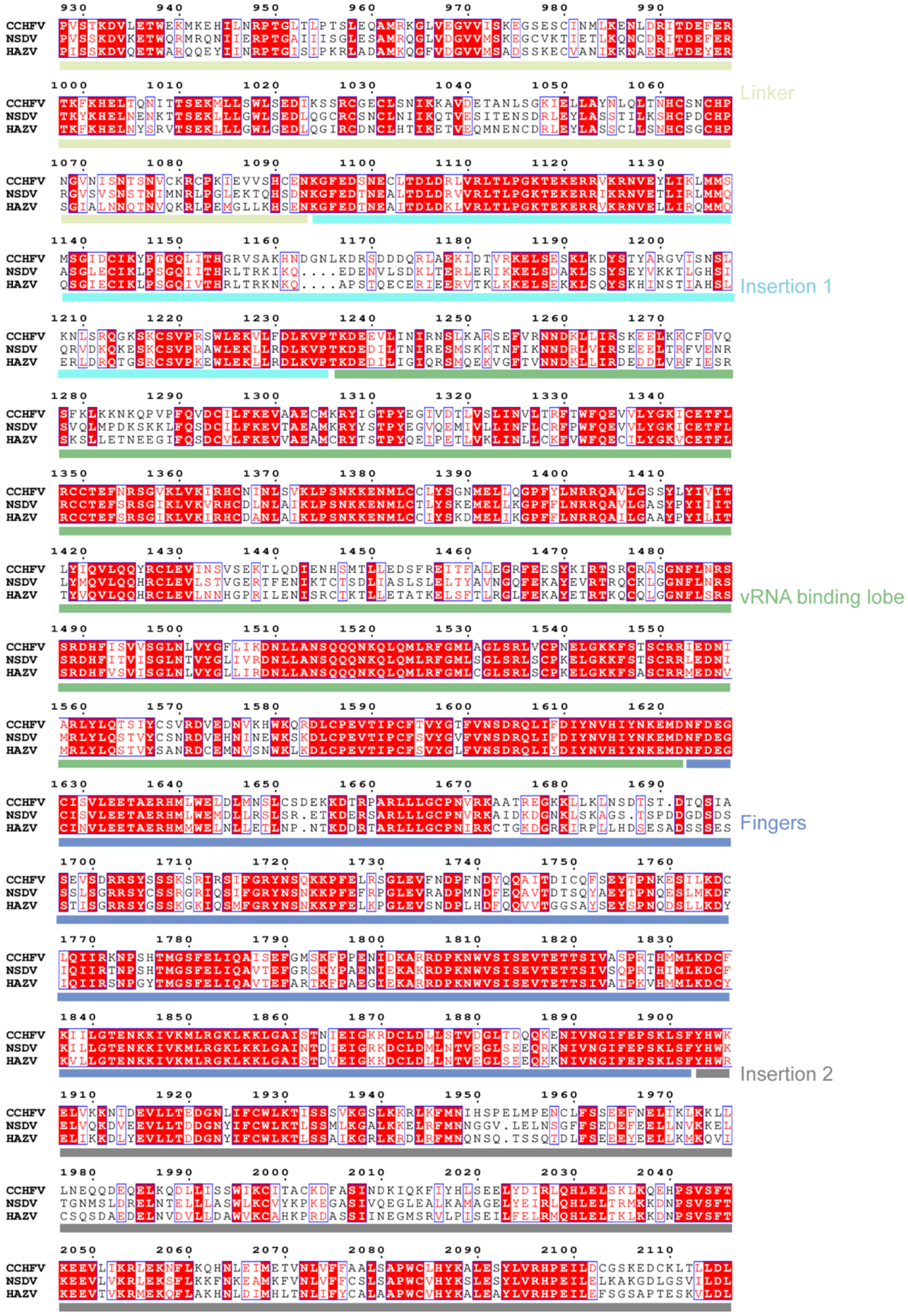

Linker
Insertion 1
vRNA binding lobe
Fingers
Insertion 2

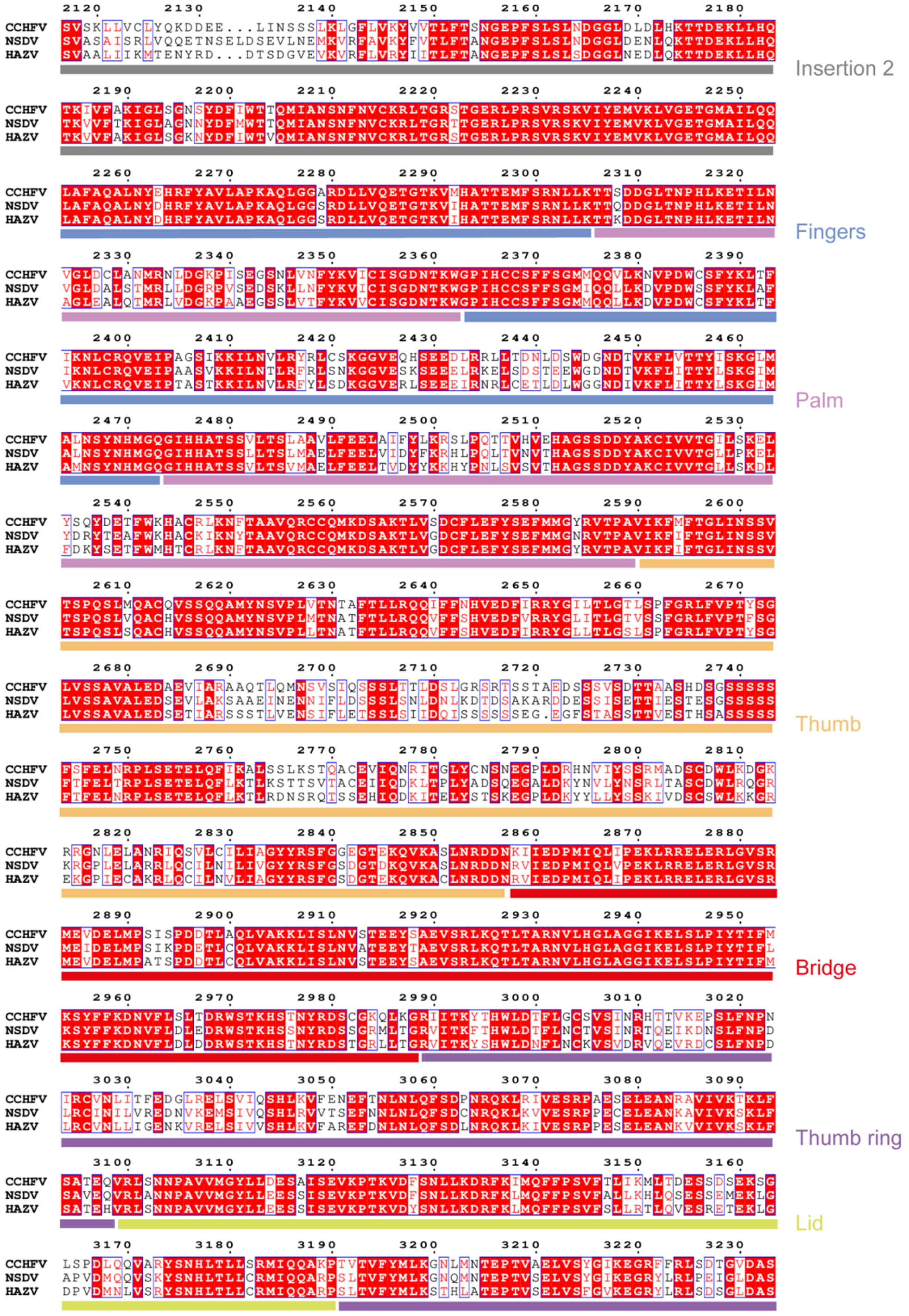

Supplementary figure 3. **Sequence alignment for nairovirus L-protein core**.
Sequence alignment for Crimean-Congo Haemorrhagic Fever Virus (Uniprot Q6TQR6), Nairobi sheep disease virus (Uniprot D0PRM7), and Hazara virus (Uniprot A6XA53) L-protein's. Domains have been annotated above the sequences.

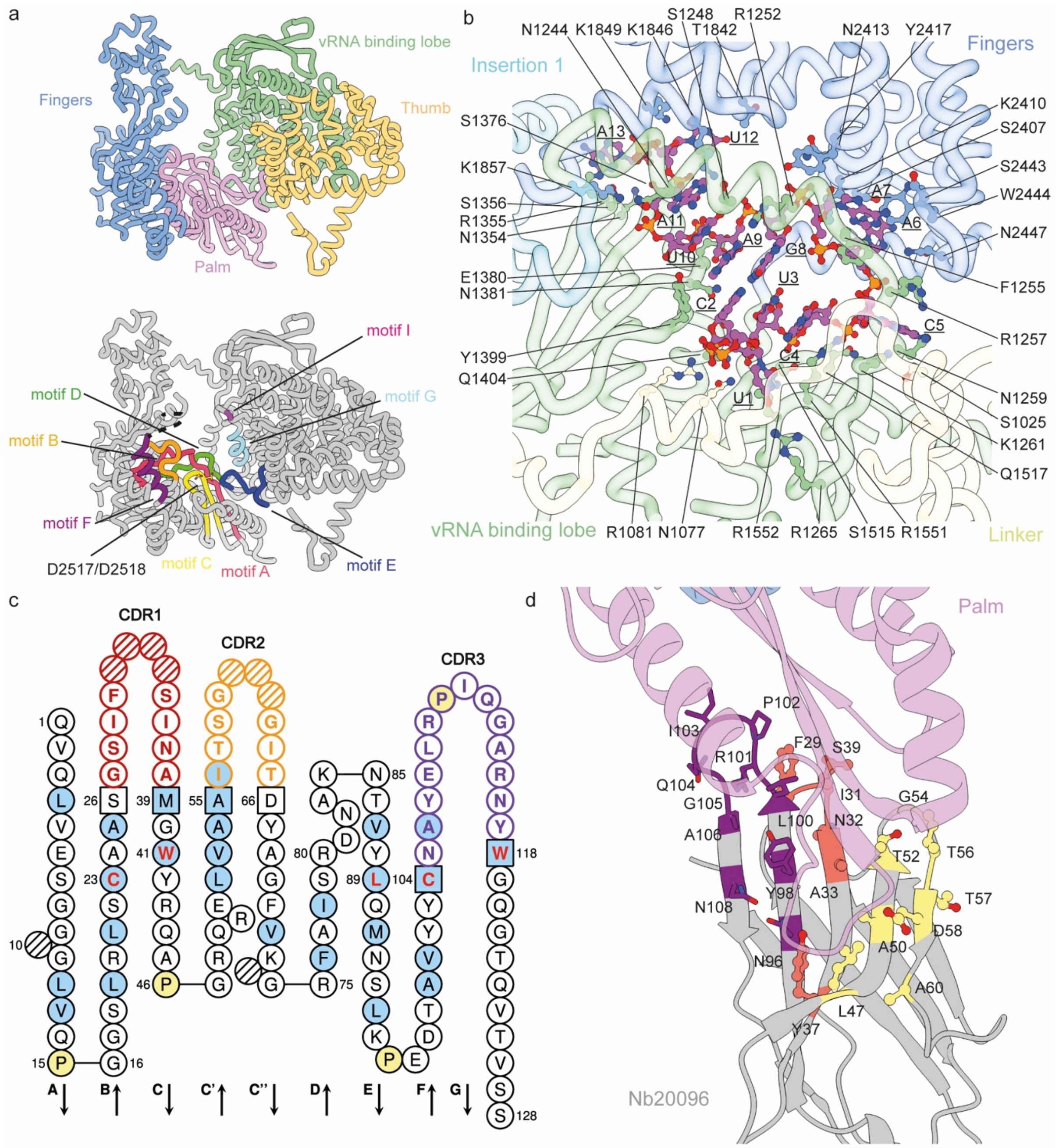

Supplementary figure 4. CCHFV-L RNA and NTP binding motifs and Nb20096 binding site. a/b) The RdRp has been annotated to show the palm (pink), thumb (yellow), vRNA binding lobe (green), and fingers (blue) sub domains. Motifs A-G and the RdRp active site residues are highlighted. b) Detailed molecular model showing the residues important in coordinating the RNA. Residues are shown in sticks and coloured according to the respective CCHFV-L domain colour code. Nucleotides are underlined and numbered. c) Amino acid sequence and topology of the Nb20096. Complementarity determining regions (CDR) are annotated. d) The interaction site between the CCHFV-L palm and Nb20096 are shown. Residues from Nb20096 that interact with the palm (pink) are annotated and coloured accordingly: CDR1 (orange), CDR2 (yellow), and CDR3 (purple).

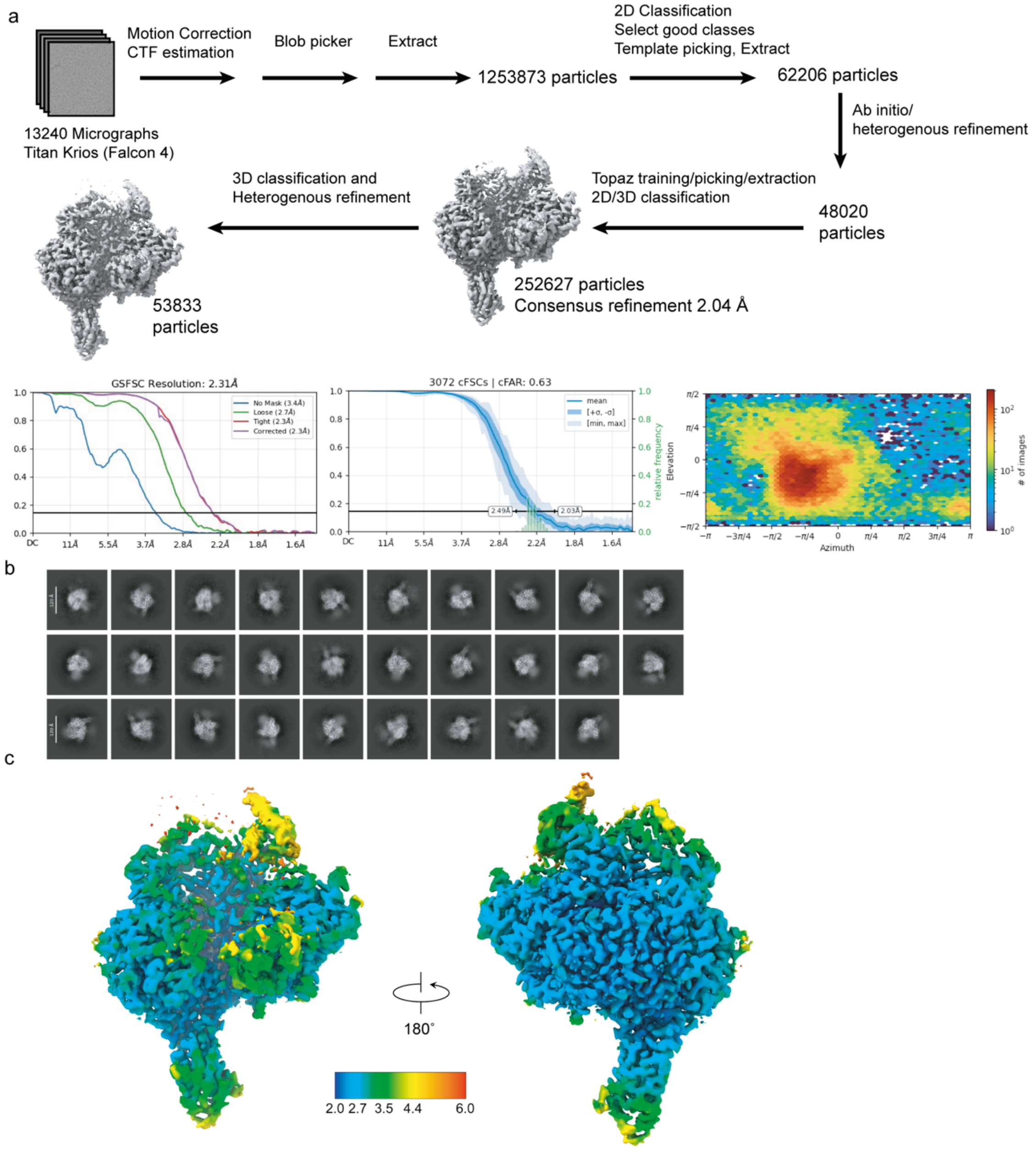

Supplementary figure 5. **RNA Bound CCHFV-L processing scheme.** a) Processing scheme for the RNA free CCHFV-L. b) 2D classes of CCHFV-L. c) Resolution range of the resulting map.

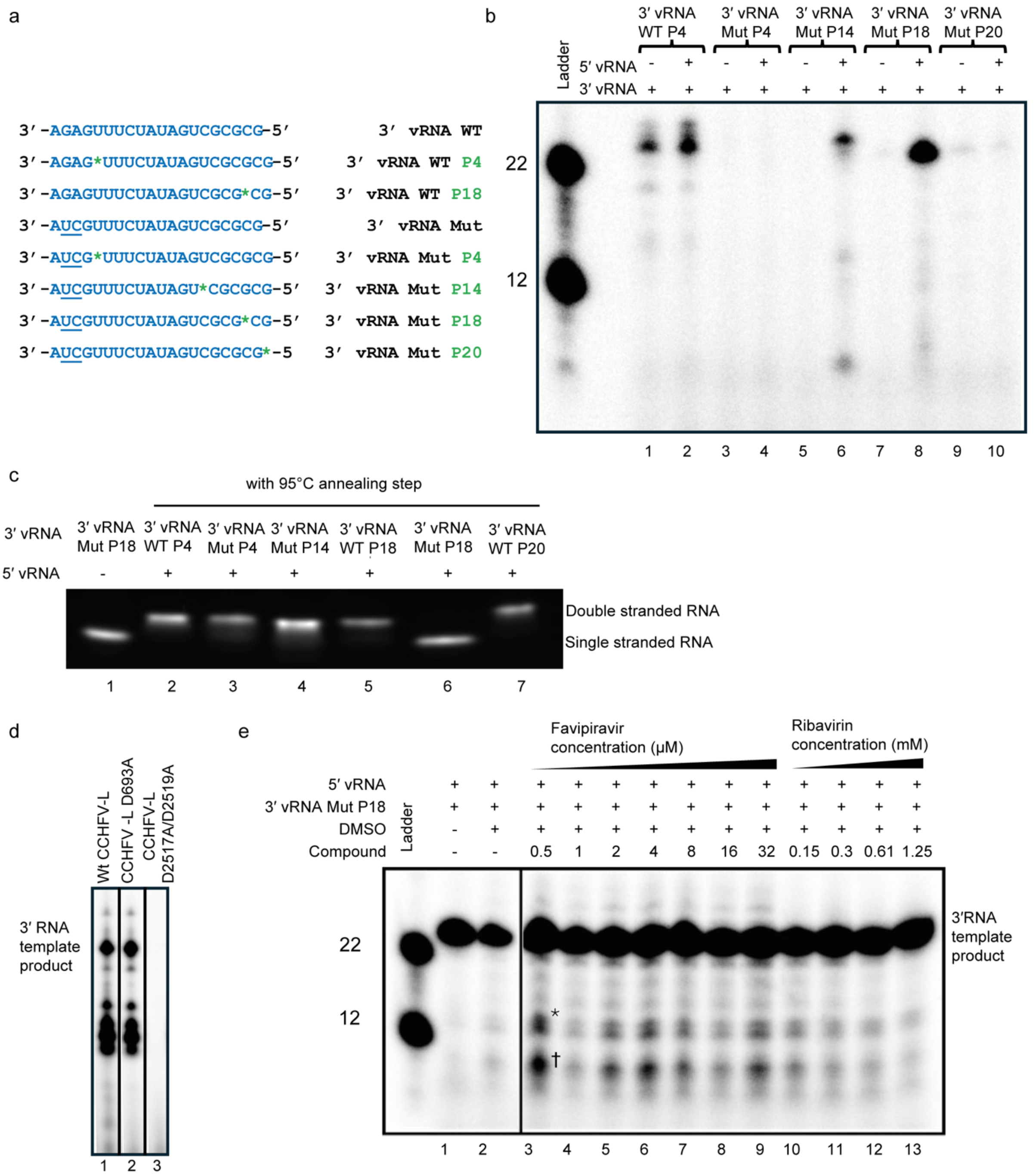

Supplementary figure 6. 32p Incorporation showing activity on different templates and in the presence of compounds. a) Scheme for 3′ vRNA templates assayed under ^{32}P-radiolabelled and fluorescent in vitro conditions. b) ^{32}P-radiolabelled reaction products generated from the fluorescent 3′ vRNAs in the presence and absence of 5′ vRNA. c) An electromobility shift assay of RNA products generated after the annealing of various fluorescently labelled 3’ vRNA to an unlabelled 5’ WT vRNA (also used in Figures 5b, 5d, 7b and 7d). Single-stranded 3’ vRNA Mut P18 was run alongside annealed products (lane 1). d) ^{32}P-radiolabelledreaction products generated from assays containing 3′ vRNA Mut P18, 5′ vRNA, and 5 nt P1-Cy5 primer using wild-type CCHFV-L, CCHFV-L D693A, and CCHFV-L D2517A/D2518A, respectively. e) Reaction products of ^{32}P-radiolabelled assays featuring the 3′ vRNA Mut P18 and 5′ vRNA in the presence of Favipiravir-TP and Ribavirin-TP. Favipiravir-TP concentration titrated from 0.5 to 32 μM, whilst Ribavirin-TP was titrated from 150 μM to

1.25 mM into the extension assays. Compound titration was performed in duplicate with similar results. * A truncated extension product produced due to the incorporation of Favipiravir or Ribavirin at positions 14-15 in the RNA transcript, resulting in the stalling and abortion of transcript extension. † A shorter truncated extension product at 5-9nt in length, produced via incorporation of Favipiravir or Ribavirin at positions 5-9 in the extension product, also resulting in stalling and abortion of extension.

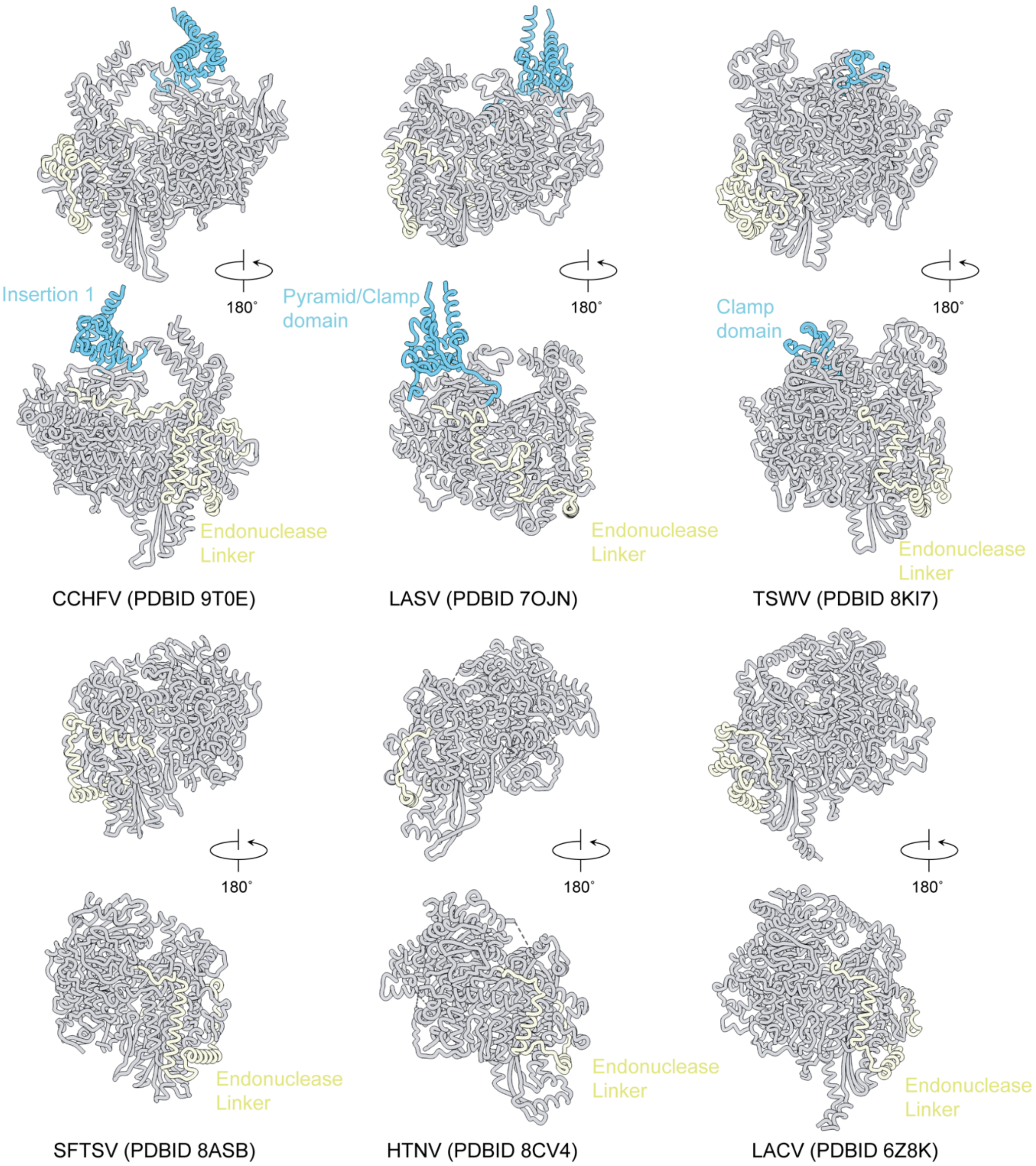

Supplementary figure 7. **Structural comparison of bunyavirus RdRp.** Representatives of the bunyavirus structures which have been determined showing the position of the core RdRp region (grey), insertions/clamp/pyramid domains (cyan), and endonuclease linker (beige). PDB ID's are annotated in the figure.
